# Supplementary material for: Brain Regeneration in Drosophila Involves Comparison of Neuronal Fitness
Source: Curr Biol. 2015 Mar 30;25(7):955–63. doi: 10.1016/j.cub.2015.02.014 (PMC4386028; doi:10.1016/j.cub.2015.02.014)
Supplement: Document S1. Supplemental Experimental Procedures and Figures S1–S3 [file mmc1.pdf]

Current Biology
Supplemental Information

Brain Regeneration in *Drosophila* Involves Comparison of Neuronal Fitness

Eduardo Moreno, Yuniel Fernandez-Marrero, Patricia Meyer, and Christa Rhiner

Supplemental Data

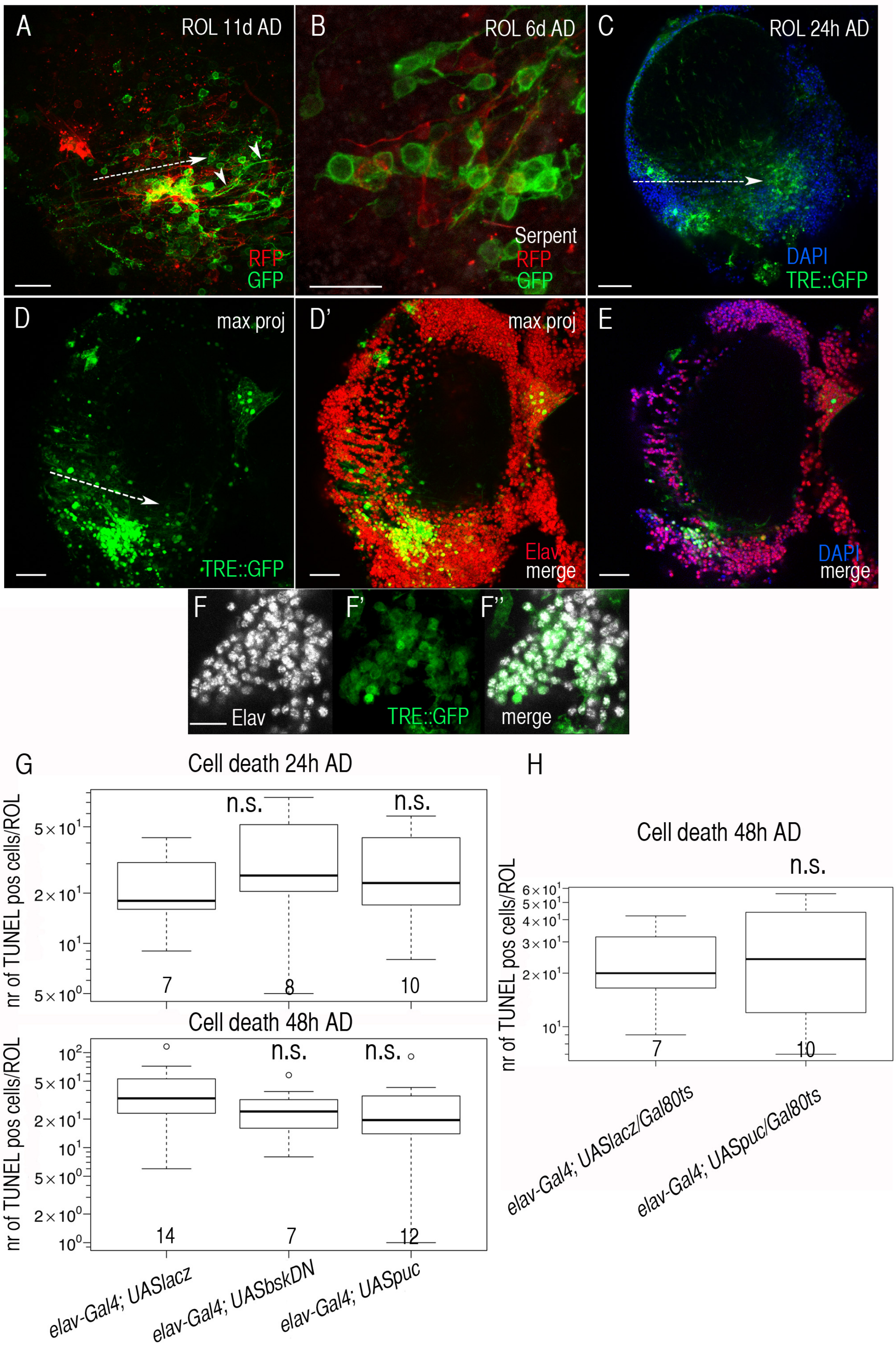

Figure S1

Figure S1, related to Figure1: Inhibition of JNK activity does not prevent cell death after brain injury

(A) Newly generated tissue marked by perma-twin labeling (GFP and RFP) shows neuronal morphology 11 days after damage (AD) in the right optic lobe (ROL). Neurons extend long axons (arrowheads) to proper target area (lobular plate). Dashed arrow marks lesion site.

(B) Perma-twin labeled tissue (GFP/RFP) does not express the hemocyte marker Serpent (white) 6 days AD [S1].

(C) JNK signaling is activated in large areas of the injured right optic lobe (ROL) based on *TRE::gfp* reporter activity.

(D-F) JNK pathway activity is present in numerous neurons 24h AD. Panels D show projections of numerous layers, E and F (inset) represent single layers. Neurons are stained with Elav (red, D, E and white, F); cell nuclei are shown in blue (E).

(G) Quantification of cell death 24h and 48h AD. JNK activity was suppressed in neurons throughout development. No statistical significance based on ANOVA (n.s.) (48h AD: $p = 0.35$ for *UASbsk*^{DN}*;* $p = 0.17$ for *UASpuc*). *$p < 0.1$.

(H) Quantification of cell death 48h AD. Inhibition of JNK activity with *UASpuc* was activated specifically in the adult nervous system. No statistical significance based on ANOVA (n.s.) ($p = 0.98$ for *UASpuc*). Bold lines show median, boxed area represents 25 and 75% quantiles. Note the logarithmic scale. n = nr of optic lobes are plotted below. Scale bars are 10 µm (B, F) and 20 µm for A, C-E).

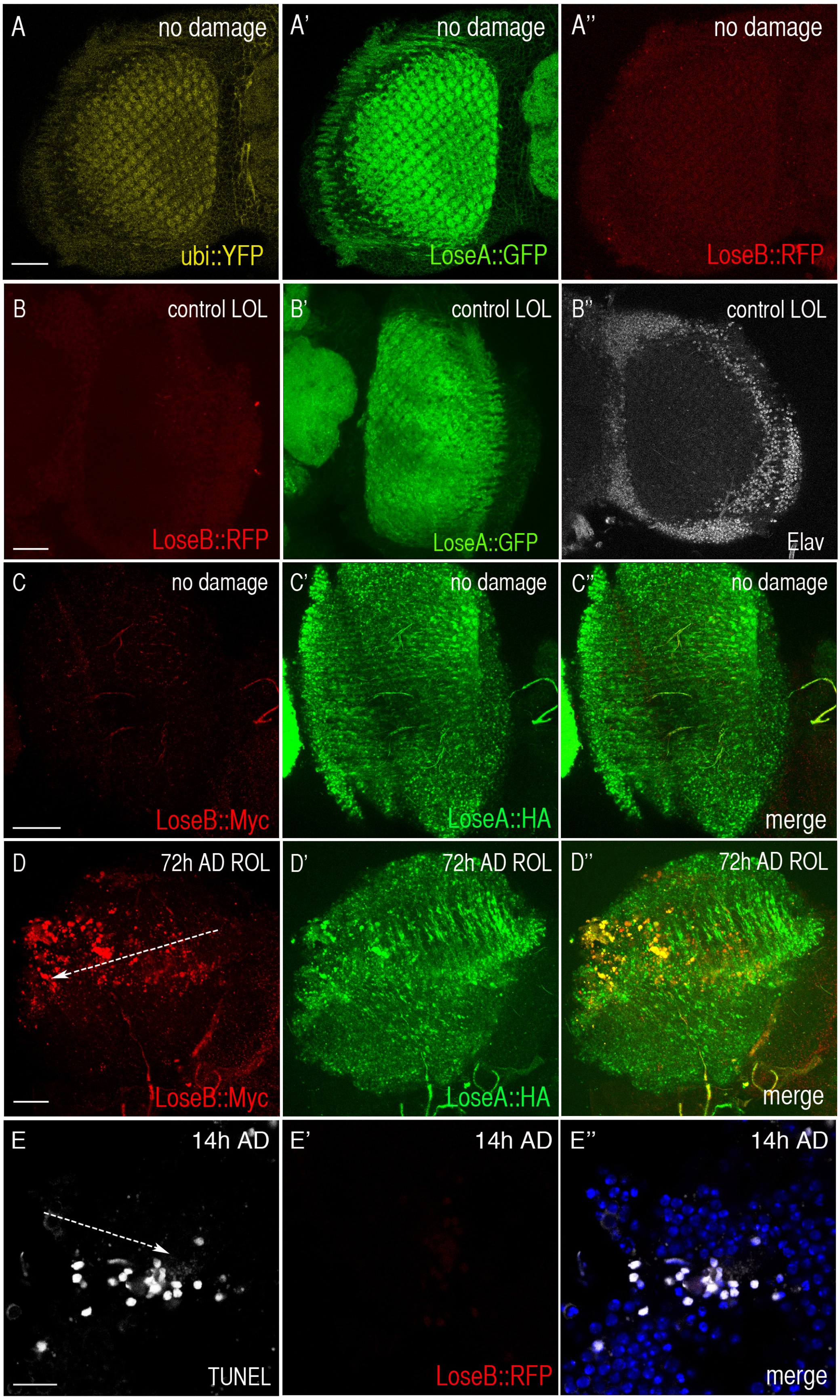
A
no damage
ubi::YFP
A'
no damage
LoseA::GFP
A''
no damage
LoseB::RFP
B
control LOL
LoseB::RFP
B'
control LOL
LoseA::GFP
B''
control LOL
Elav
C
no damage
LoseB::Myc
C'
no damage
LoseA::HA
C''
no damage
merge
D
72h AD ROL
LoseB::Myc
D'
72h AD ROL
LoseA::HA
D''
72h AD ROL
merge
E
14h AD
TUNEL
E'
14h AD
LoseB::RFP
E''
14h AD
merge

Figure S2

Figure S2, related to Figure 2: Damage-modulated expression of Flower isoforms

(A) Expression of $Flower^{ubi}$ (YFP), $Flower^{LoseB}$ (RFP) and $Flower^{LoseA}$ (GFP) in the optic lobe of the adult brain based on a translational *flower* reporter.

(B) Expression of $Flower^{LoseB}$ (RFP) and $Flower^{LoseA}$ (GFP) in the undamaged control left optic lobe (LOL). Neurons (Elav+) are shown in white.

(C and D) Flower isoform expression in undamaged (C) and apically damaged optic lobes (arrow, through head cuticle) (D) as evidenced by antibody staining against HA-tagged $Flower^{LoseA}$ (green) and Myc-tagged $Flower^{LoseB}$ (red) based on a translational *flower* reporter 72h after damage (AD).

(E) Apoptotic cells (TUNEL, white) 14h AD do not show upregulation of $Flower^{LoseB::RFP}$ (red). Arrow marks injury site.

Scale bars are 20 μm (A-D) and 10 μm (E).

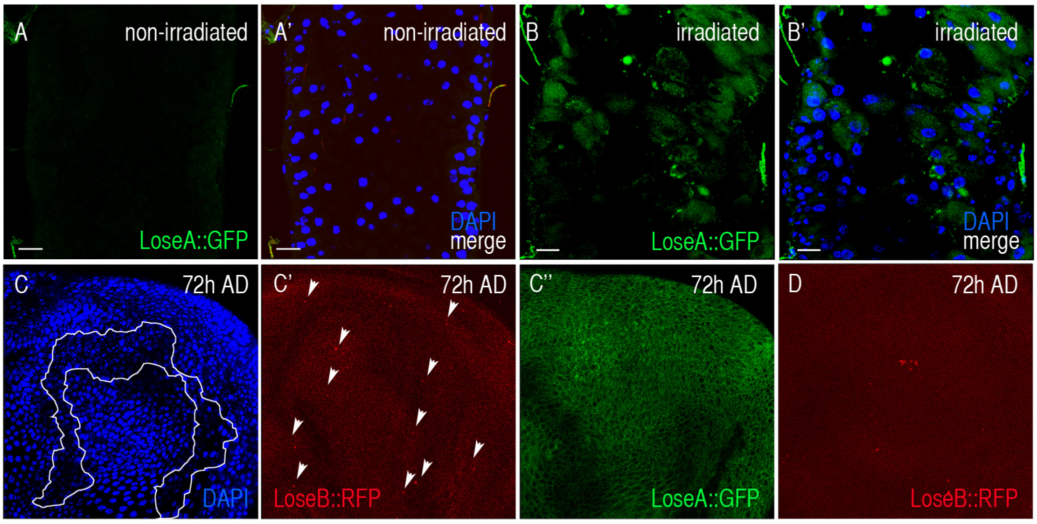

Figure S3

Figure S3, related to Figure 2: Flower fitness fingerprints in the regenerating gut.

(A) Flower^{LoseA} (GFP) expression in the non-irradiated midgut. Nuclei are shown in blue (DAPI).

(B) Flower^{LoseA} (GFP) is upregulated in the midgut 24h after irradiation.

(C) Flower^{LoseB} expression (RFP, arrowheads) 72h after wing disc ablation with *rnGal4, UASeiger, tubGal80^{ts}* in the apoptotic region (marked in white) containing numerous apoptotic corpses (small nuclei, DAPI, blue).

(D) Foci of Flower^{LoseB}-expressing cells (RFP) 72h after wing disc ablation with *rnGal4, UASeiger, tubGal80^{ts}*.

Scale bars are 20 μm.

Supplemental Experimental Procedures

Midgut irradiation. 2-3 days old adult flies were subjected to 2 x 10^{-2} J of UV irradiation with a UV Stratalinker 2400 machine and analyzed for Flower isoform expression 24h later.

Wing pouch ablation. *w^{1118}; +; rnGal4, UASeiger, tubGal80^{ts}* flies were crossed to *fweReporter(yfp_gfp_rfp)* flies at 18°C. Larvae were shifted for 20h to 29°C to induce wing disc ablation and then placed back to 18°C to allow regeneration.

Supplemental References

[S1] Lebestky T, Chang T, Hartenstein V, Banerjee U (2000). Specification of Drosophila hematopoietic lineage by conserved transcription factors. Science. 288:146-149.
