# Supplementary material for: Brain Regeneration in Drosophila Involves Comparison of Neuronal Fitness
Source: Curr Biol. 2015 Mar 30;25(7):955–63. doi: 10.1016/j.cub.2015.02.014 (PMC4386028; doi:10.1016/j.cub.2015.02.014)
Supplement: Document S2. Article plus Supplemental Information [file mmc2.pdf]

Report

Current Biology

Brain Regeneration in *Drosophila* Involves Comparison of Neuronal Fitness

Highlights

- Brain injury modulates neuronal fitness fingerprints in the adult brain
- Comparison of neuronal fitness drives brain tissue replacement in *Drosophila*
- De novo-generated cells are favored over damage-affected neurons

Authors

Eduardo Moreno, Yuniel Fernandez-Marrero, Patricia Meyer, Christa Rhiner

Correspondence

christa.rhiner@izb.unibe.ch

In Brief

After injury, less functional neurons are contained within the brain. Here, Moreno et al. use a *Drosophila* model of brain regeneration to show that impaired neurons are eliminated next to newly generated cells because they carry low-fitness marks. Such interactions between old and new tissues may be relevant for other regenerative processes.

CrossMark

Moreno et al., 2015, Current Biology *25*, 955–963
March 30, 2015 ©2015 The Authors
http://dx.doi.org/10.1016/j.cub.2015.02.014

CellPress

Current Biology
Report

Brain Regeneration in *Drosophila* Involves Comparison of Neuronal Fitness

Eduardo Moreno,[1] Yuniel Fernandez-Marrero,[1] Patricia Meyer,[1] and Christa Rhiner[1,*]
[1]Institute of Cell Biology (IZB), University of Bern, Bern 3012, Switzerland
*Correspondence: christa.rhiner@izb.unibe.ch
http://dx.doi.org/10.1016/j.cub.2015.02.014
This is an open access article under the CC BY license (http://creativecommons.org/licenses/by/4.0/).

SUMMARY

Darwinian-like cell selection has been studied during development and cancer [1–11]. Cell selection is often mediated by direct intercellular comparison of cell fitness, using "fitness fingerprints" [12–14]. In *Drosophila*, cells compare their fitness via several isoforms of the transmembrane protein Flower [12, 13]. Here, we studied the role of intercellular fitness comparisons during regeneration. Regeneration-competent organisms are traditionally injured by amputation [15, 16], whereas in clinically relevant injuries such as local ischemia or traumatic injury, damaged tissue remains within the organ [17–19]. We reasoned that "Darwinian" interactions between old and newly formed tissues may be important in the elimination of damaged cells. We used a model of adult brain regeneration in *Drosophila* in which mechanical puncture activates regenerative neurogenesis based on damage-responsive stem cells [20]. We found that apoptosis after brain injury occurs in damage-exposed tissue located adjacent to zones of de novo neurogenesis. Injury-affected neurons start to express isoforms of the Flower cell fitness indicator protein not found on intact neurons. We show that this change in the neuronal fitness fingerprint is required to recognize and eliminate such neurons. Moreover, apoptosis is inhibited if all neurons express "low-fitness" markers, showing that the availability of new and healthy cells drives tissue replacement. In summary, we found that elimination of impaired tissue during brain regeneration requires comparison of neuronal fitness and that tissue replacement after brain damage is coordinated by injury-modulated fitness fingerprints. Intercellular fitness comparisons between old and newly formed tissues could be a general mechanism of regenerative tissue replacement.

RESULTS

In many clinically relevant injuries, such as stroke or traumatic brain injury, impaired cells remain within an organ. In order to study how damaged brain tissue interacts and may be replaced by newly generated cells after injury, we subjected adult flies to penetrating traumatic brain injury, by lesioning the optic lobe (OL) unilaterally with a thin metal filament (Figures 1A and 1B). This local mechanical damage has been previously shown to activate quiescent adult neural stem cells and drive regenerative neurogenesis [20], therefore leading to the apposition of injury-exposed and intact neurons, as well as de novo generated neurons. Local recruitment and activation of stem cells is a common strategy to regenerate tissues in many organisms [15, 16, 21].

Traumatic brain injuries typically cause a variable extent of tissue damage. Neurons can persist in vulnerable states due to axon stretching and tearing, activating secondary injury processes (diffuse neuronal depolarization, glutamate excitotoxicity, disturbed calcium homeostasis, etc.), which are poorly understood [18, 19]. To study the fate of impaired brain tissue, we decided to monitor cell death several days after the primary injury.

Pre-existing Tissue Undergoes Apoptosis at Sites of De Novo Neurogenesis after Traumatic Brain Injury

We have previously shown that neuronal apoptosis is detectable within the first hours after damage (AD) as a direct consequence of the mechanical impact [20]. Extended analysis revealed a second burst of apoptosis starting at around 24 hr AD, with low numbers of apoptotic cells present in the lesioned area (Figure 1C), which increased and peaked around 3 days after injury (Figure 1D). To determine whether apoptosis occurred within regenerating or pre-existing tissue, we performed TUNEL staining of injured brains in which proliferating cells upon injury were marked with GFP/RFP based on perma-twin labeling [20], a mitotic recombination-dependent tracing method, which is activated before brain damage in adult *Drosophila* to mark newly generated tissue [20]. Three days after brain injury, we observed numerous apoptotic cells in damage-exposed tissue next to new tissue (Figures 1E). Even 6 days AD, cells continued to die in the "old" tissue neighboring patches of regenerated tissue (Figures 1F and 1G), whereas undamaged OLs did not show apoptosis associated with newly generated cells (n = 20 OLs) derived from physiologic adult neurogenesis (Figure 1H) [20].

The newly formed tissue observed 6 days after brain damage consisted mainly of newborn neurons (Figure 1I) [20], which expressed the panneuronal marker Elav [22] and persisted up to 11 days AD (Figure S1A). Regenerated tissue was usually devoid of glial cells [20] and macrophages (Figure S1B).

Most apoptotic cells were found close (within three cell diameters) to newly generated cells 3 days and 6 days AD (81% and 90% of total cell death, respectively) (Figure 1J). In contrast, apoptosis rarely occurred in "perma-twin-marked" new tissue

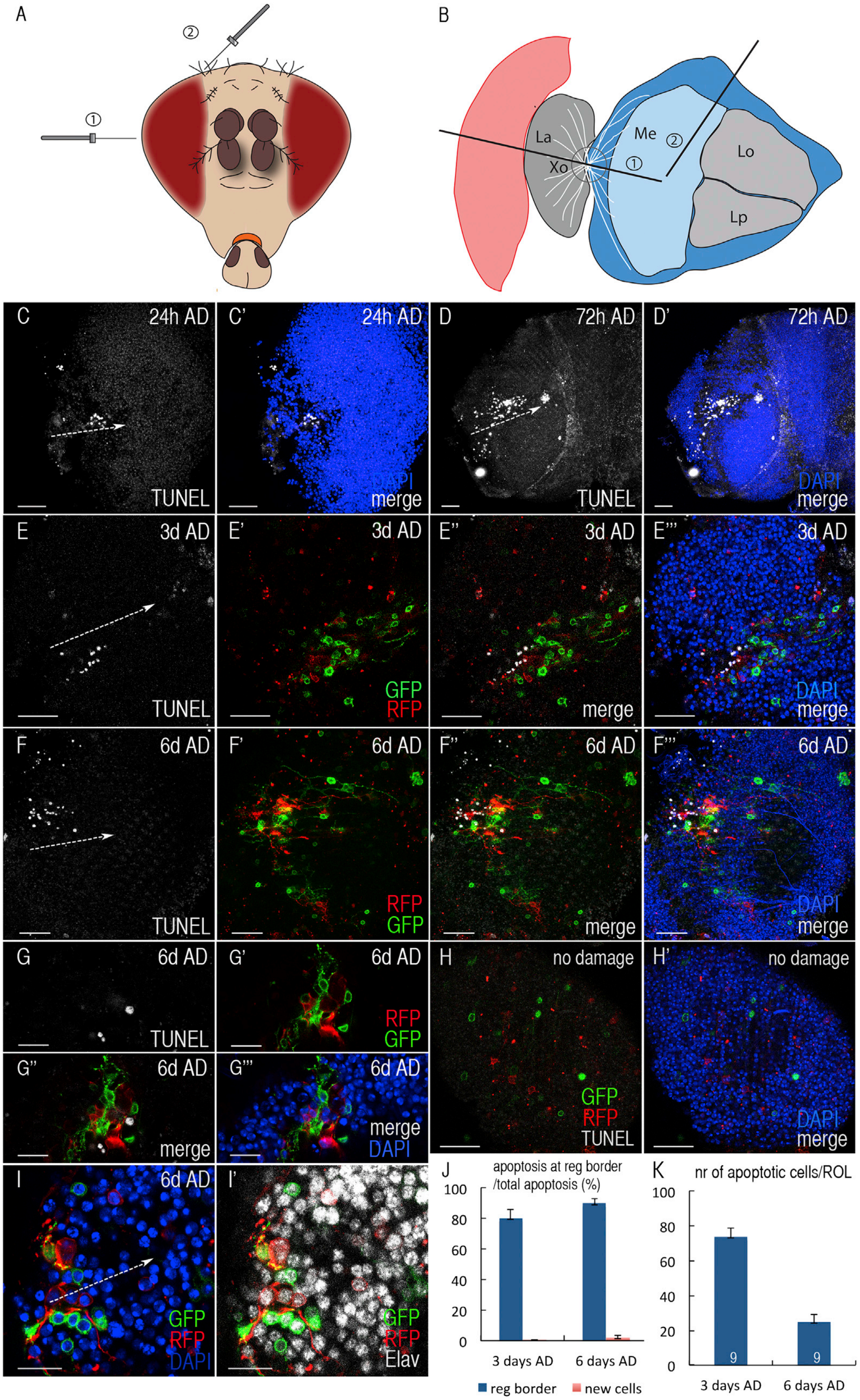

(legend on next page)

(0.5% of total cell death at 3 days AD and 2% ± 2% at 6 days AD) (Figure 1J). Overall, apoptotic counts were highest 3 days AD (74 ± 13 apoptotic cells/OL) and dropped to one-third around 6 days after injury (25 ± 11 cells/OL), accompanied by a proliferative phase, evident from the expansion of perma-twin-marked tissue (Figures 1E, 1F, and S1A and as shown previously [20]).

Thus, we have identified a burst of delayed cell death in injury-exposed brain tissue that is not caused by the primary mechanical insult but is associated with the onset of regenerative neurogenesis.

Adult Neurons Express Fitness Indicator Proteins

In order to find genes that may regulate cell death at regeneration borders, we tested reporters for pathways such as JNK [23, 24], Hippo [25–27], Wingless [28, 29], and JAK-STAT [30] that are important for regeneration of fly epithelial tissues. Among these, only *TRE-gfp*, a sensitive JNK pathway reporter [31], was strongly induced after brain damage (Figures S1C–S1F).

We repressed JNK signaling in neurons during all stages or specifically during adulthood, but we did not observe any significant reduction of cell death 1 or 2 days after brain injury (Figures S1G and S1H).

Next, we hypothesized that "Darwinian-like" interactions between impaired and newly formed tissues may trigger cell death, since negative selection can drive elimination of less fit cells during development or carcinogenesis [1–11].

In *Drosophila*, different isoforms of the conserved Flower protein form tissue-specific fitness fingerprints at the cell surface (Figure 2A) that mediate negative selection of suboptimal cells when surrounded by fitter cells [12, 13]. First, we asked whether Flower isoforms are expressed in the adult brain and, specifically, in the OLs. To this end, we used transgenic flies carrying a translational *flower* reporter in which expression of $Flower^{ubi}$, $Flower^{LoseA}$, and $Flower^{LoseB}$ can be visualized as fusion proteins to YFP, GFP, and RFP, respectively [32]. $Flower^{LoseA::GFP}$ was strongly expressed in the adult brain, including the OLs, whereas $Flower^{LoseB::RFP}$ was not detectable (Figure 2B). Since $Flower^{ubi::YFP}$ signal was of low intensity (Figure S2A), we verified the expression pattern of $Flower^{ubi}$ with an ubi-specific antibody [12]. We found that both $Flower^{LoseA}$ and $Flower^{ubi}$ localized to cell membranes (Figures 2B and 2C), but $Flower^{ubi}$ levels were lower since immunodetection required signal amplification. Next, we stained adult brains for Elav, which showed that mature neurons in the adult brain display $Flower^{LoseA}$ and low levels of $Flower^{ubi}$ at the cell surface (Figure 2D and data not shown). This revealed that Flower proteins are not only expressed during nervous system development [13], but also form similar fitness fingerprints in the adult nervous system.

Brain Injury Modulates the Expression of Fitness Indicator Proteins

Subsequently, we injured OLs unilaterally and observed $Flower^{LoseB::RFP}$ induction specifically in the damaged right OLs (Figures 2E–2G) compared to the undamaged control side (Figure S2B). $Flower^{LoseB::RFP}$ signal was first detectable in few cells 24 hr AD and was then present in numerous cells along the lesion 48 and 72 hr AD, whereas expression levels of $Flower^{ubi}$ and $Flower^{LoseA}$ remained similar (Figures 2E–2G). Next, we repeated the experiments with a different *flower* reporter where the three isoforms carry Flag ($Flower^{ubi}$), HA ($Flower^{LoseA}$), and Myc ($Flower^{LoseB}$) tags [32]. Again, we found that $Flower^{LoseB::Myc}$ was upregulated in lesioned OLs compared to uninjured brains, whereas $Flower^{LoseA::HA}$ was expressed at high levels in damaged and undamaged OLs (Figures S2C and S2D).

Elav staining of flies carrying the *flower* reporter revealed that $Flower^{LoseB::RFP}$ was induced at the cell surface of injury-exposed neurons 48 hr AD (Figure 2H, arrowheads) or present in dying neurons ($Elav^+$) (Figure 2H, arrow).

These results show that acute brain injury triggers local and dynamic changes in displayed fitness marks on damage-exposed neurons compared to surrounding, non-affected cells: impaired neurons start to signal low fitness via induction of $Flower^{LoseB}$, which is not encountered on healthy neurons, whereas $Flower^{LoseA}$ and $Flower^{ubi}$ expression remains comparable on injured versus non-injured cells (Figure 2I).

$Flower^{LoseB}$ Expression Is Associated with Cell Death

In order to relate $Flower^{LoseB}$ induction to cell death during brain regeneration, we performed TUNEL staining of flies transgenic for the YFP/GFP/RFP translational *flower* reporter. We observed that $Flower^{LoseB}$ expression often correlated with cell death 1 to 3 days after brain injury (Figures 3A and 3B). At 72 hr after injury, 64% ± 9% of $Flower^{LoseB::RFP}$-expressing cells stained positive for TUNEL (n = 11 OLs), raising the possibility that $Flower^{LoseB}$ expression could drive negative neuronal selection, as previously described for neuronal culling during retina development [13].

Interestingly, $Flower^{LoseB}$ was not detected in apoptotic cells 6–14 hr after mechanical injury (Figure S2E), suggesting that immediate cell death after mechanical tissue disruption may be $Flower^{LoseB}$ independent.

Figure 1. Cell Death during Tissue Regeneration

(A and B) *Drosophila* head (frontal view) indicating the site of traumatic brain injury either via eye (lateral; 1) or head cuticle (apical; 2) (A) and corresponding needle paths (B). Xo, outer optic chiasm; Me, Medulla; La, lamina; Lo, lobula; Lp, lobular plate.

(C and D) Cell death (white; TUNEL) 24 hr (C) and 72 hr (D) after brain damage (AD). Cell nuclei are shown in blue (DAPI).

(E–G) Apoptosis (TUNEL; white) in laterally injured OLs 3 days (E) and 6 days (F and G) AD. Regenerated tissue is marked by perma-twin labeling (GFP/RFP), and nuclei are shown in blue (DAPI). Inset shows cell death (TUNEL, white) near regenerated cells (GFP/RFP) (G).

(H) TUNEL staining (white) of undamaged control OLs. Physiologic adult neurogenesis in the medulla cortex of the OLs is revealed by perma-twin tracing (GFP/RFP). DAPI marks cell nuclei (blue).

(I) Regenerated tissue (perma-twin tracing, GFP/RFP) 6 days AD consists of $Elav^+$ neurons (white); DAPI marks nuclei (blue).

(J) Graph showing percentage of TUNEL-positive cells at the regeneration border (maximum of three cell diameters away from new tissue; blue bars) and in regenerated cells (new cells; red bars) 3 days and 6 days AD. Error bars indicate the SD. n = 9 right OLs.

(K) Graph depicting the number (nr) of apoptotic cells per damaged right OL (ROL) in perma-twin flies 3 days and 6 days AD. Error bars indicate the SEM. Numbers indicate n.

Scale bars represent 10 μm (G and I) and 20 μm (C–F and H). Dashed arrows (C–F and H) mark the area of injury. See also Figure S1.

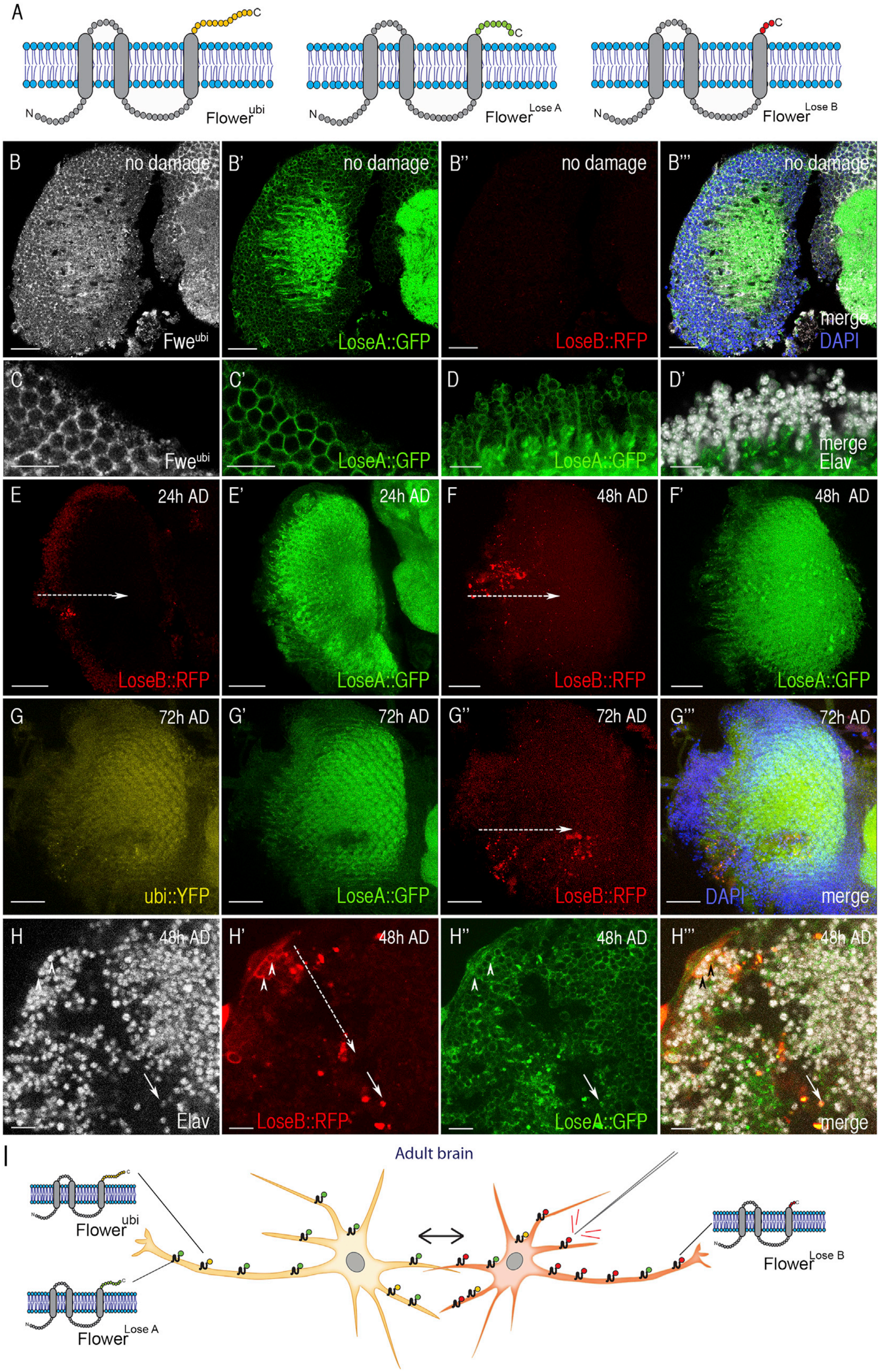

(legend on next page)

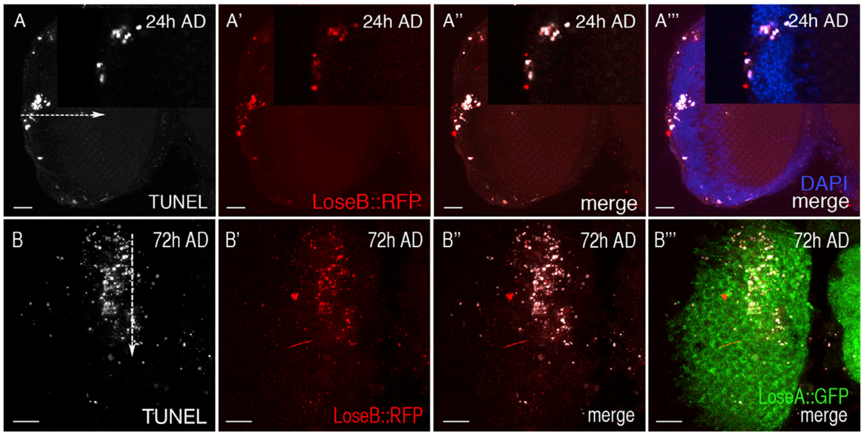

Figure 3. Flower^{LoseB} Is Upregulated in Apoptotic Cells

(A) A subset of apoptotic cells (TUNEL; white) in laterally injured OLs (arrow) express Flower^{LoseB} (red) 24 hr AD. Cell nuclei are stained with DAPI (blue). The upper-right panels show an inset of the same right OL.

(B) Apoptotic cells in apically injured OL (through cuticle; arrow) express Flower^{LoseB} 72 hr AD. Ubiquitous Flower^{LoseA} expression is shown in the merged image (green).

Scale bars represent 20 μm.

Flower Fitness Marks Mediate Negative Selection of Unfit Neurons during Brain Regeneration

To examine whether Flower is functionally implicated in neuronal cell death linked to *flower^{LoseB}* upregulation, we conditionally activated *UASflowerRNAi* constructs in the adult nervous system using the neuronal driver *elav-Gal4* and the thermosensitive Gal4 repressor Gal80^{ts}. Five days after gene activation, OLs were lesioned laterally and brains were processed for TUNEL staining.

Three days after injury, knockdown of all *flower* isoforms (*UASRNAifwe_all*) or both *flower* Lose isoforms (*UASRNAifweLA/LB*) in adult neurons significantly reduced apoptosis in damaged right OLs (12 ± 4 dead cells/right OL) compared to control brains (87 ± 22 cells/right OL), where expression of *UASlacz* was activated instead (ANOVA: $p = 9.7 \times 10^{-14}$ for *UASRNAifwe_all* and $p = 1.1 \times 10^{-13}$ for *UASRNAiLoseA/LoseB*) (Figures 4A–4D and 4J). Apoptotic numbers were already reduced 24 hr AD when Flower fitness fingerprints were suppressed by the same RNAi constructs (ANOVA: $p = 7.7 \times 10^{-6}$ for *UASRNAifwe_all* and $p = 2.4 \times 10^{-5}$ for *UASRNAiLoseA/LoseB*) (Figure 4I).

Sequence similarities between *flower^{LoseA}* and *flower^{LoseB}* mRNAs did not allow *flower^{LoseB}*-specific targeting. These results show that Flower fitness indicator proteins are functionally required in neurons to signal removal of unfit neurons.

Cell Death after Traumatic Brain Injury Is Regulated through Neuronal Fitness Comparison

Since Flower isoforms have previously been shown to reveal fitness deficits in a non-cell-autonomous manner [12, 13], we tested whether uniform overexpression of low fitness marks (in this case Flower^{LoseB}) would prevent apoptosis coinciding with brain regeneration. To this end, we activated overexpression of *UASflower^{LoseB}*, *UASflower^{LoseA}*, and *UASflower^{ubi}* in all neurons in adult flies and examined the effect on cell death (Figures 4B and 4E–4J).

When *flower*^{LoseB} was ectopically expressed in adult brains, apoptotic counts in lesioned brains were halved 24 hr AD compared to *UASlacz* control brains (ANOVA $p = 2.4 \times 10^{-5}$) (Figure 4I) and ten times lower at the third day after traumatic brain injury (9 ± 4 cells/right OL; ANOVA: $p = 1.9 \times 10^{-14}$) (Figures 4G and 4J), whereas uninjured left OLs (*UASlacz*) showed on average 4 ± 1 apoptotic cells (Figures 4H and 4J). In contrast, neuronal overexpression of *flower^{LoseA}* and *flower^{ubi}* did not significantly affect the number of TUNEL-positive cells 24 and 72 hr after brain injury (ANOVA: $p \geq 5.1 \times 10^{-1}$) (Figures 4I and 4J).

These results show that the majority of cell death 3 days after traumatic brain injury is actively regulated through comparison of neuronal fitness, leading to elimination of Flower^{LoseB}-expressing impaired neurons when surrounded by intact or newly formed neurons with more advantageous fitness profiles (Figure 4K).

DISCUSSION

Darwinian-like cell selection plays an important role when constructing tissues during development [1–5, 7, 8]. Here, we have addressed how the brain weeds out less functional neurons after injury. We show that fitness-based cell selection regulates the elimination of damaged tissue during adult brain regeneration in *Drosophila*. Based on reporter screening and genetic

Figure 2. Brain Injury Modulate Fitness Fingerprints of Adult Neurons

(A) Different isoforms of the cell membrane protein Flower (ubi, LoseA, and LoseB) form tissue-specific fitness fingerprints. Identical regions are depicted in gray.

(B) Expression of Flower^{LoseA} and Flower^{LoseB} in intact OLs in green and red, respectively. Staining for Flower^{ubi} is shown in white. DAPI marks nuclei (blue).

(C and D) Insets show Flower^{ubi} (C) and Flower^{LoseA} expression (C and D) on mature neurons (Elav^{+}; white) (D).

(E and F) *Flower^{LoseB}* (red) induction in laterally punctured OLs 24 hr and 48 hr AD.

(G) Flower^{LoseB} expression (red) in laterally injured OLs 72 hr AD.

(H) At 48 hr after injury, Flower^{LoseB} (red) is upregulated in injury-exposed neurons (Elav^{+}; white). Arrowheads point to neurons with induced Flower^{LoseB} expression, and the arrow shows nuclear accumulation of Flower^{LoseB} in a dying neuron (small nucleus; Elav^{+}). Flower^{LoseA} (green) shows panneuronal expression.

(I) Scheme illustrating the change in Flower fitness fingerprints upon damage.

Dashed arrows mark needle insertion sites. Scale bars represent 10 μm (C, D, and H) and 20 μm (B and E–G). See also Figures S2 and S3.

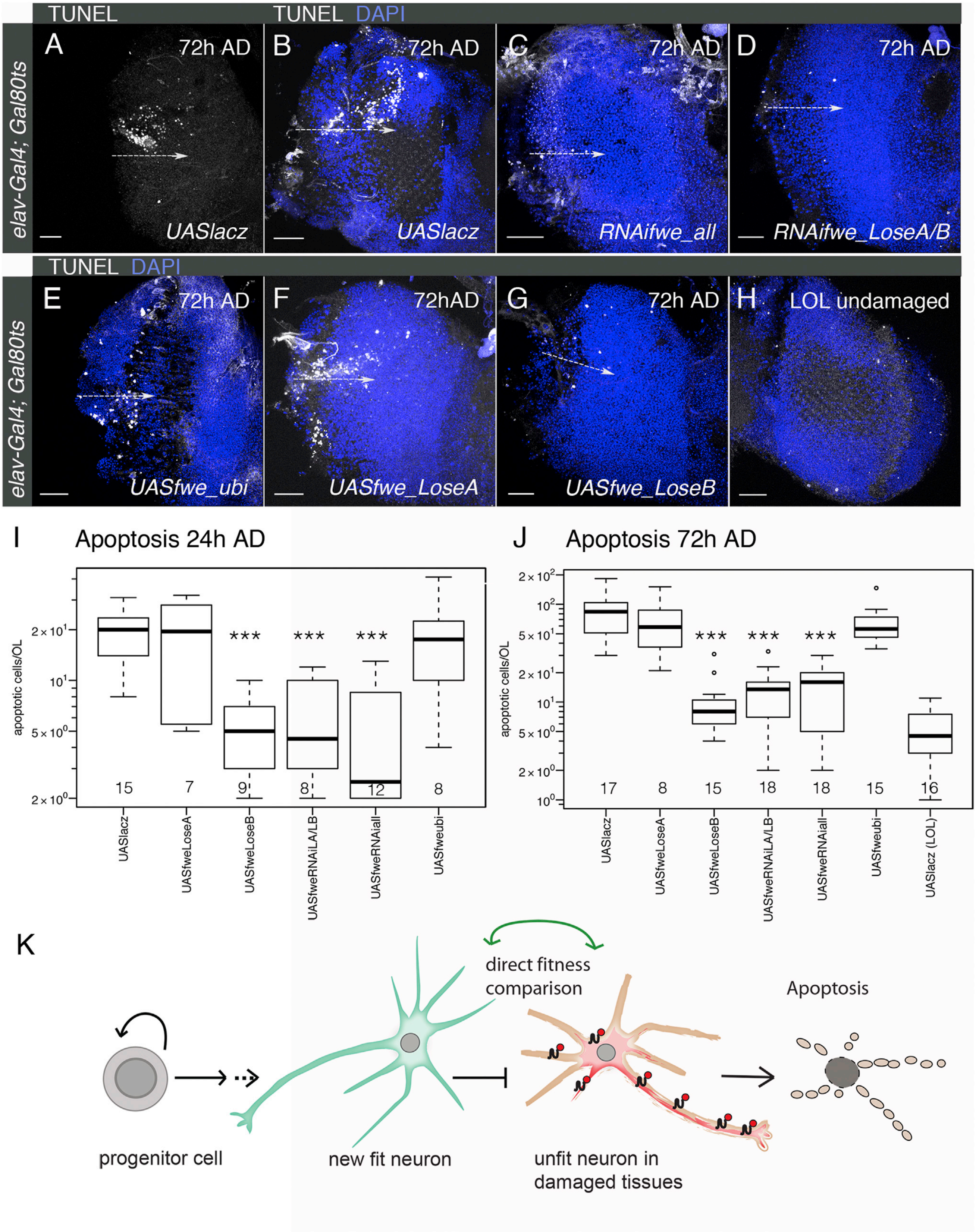

(legend on next page)

analyses, we have found that specific isoforms of the cell fitness indicator protein Flower drive the active elimination of impaired neurons at stages in which regenerative neurogenesis provides new neurons for repair.

We could show that traumatic brain injury causes fitness deficits in injury-exposed neurons, which start to express $\text{Flower}^{\text{LoseB}}$ isoforms that are absent on healthy neurons (Figure 2). This reveals for the first time that fitness indicator proteins operate in the adult nervous system and are able to dynamically reflect changes in neuronal fitness states. Local $\text{Flower}^{\text{LoseB}}$ upregulation also mediates the culling of sensory neurons in incomplete photoreceptor units during development [13] and therefore seems to present a common signal to mediate negative neuronal selection. These findings open the possibility that conserved Flower proteins may reflect changes in neuronal fitness in other neuropathological conditions.

For further insight, it will be helpful to determine which unfit neurons are recognized and selected for replacement. $\text{Flower}^{\text{LoseB}}$ could be upregulated upon physical damage to the neuronal cell body, axon shearing, or disruption of proper wiring or a combination of insults. Moreover, for damaged brain tissue to be replaced, not only neuronal cell bodies but also their axonal projections need to be removed efficiently. It is therefore possible that $\textit{flower}^{\textit{LoseB}}$ induction and "axon death" signaling molecules [33], which trigger Wallerian degeneration to allow fast fine-tuning of the nervous system, may be linked.

Importantly, we show that damage-modulated fitness indicator proteins are necessary to identify and cull impaired neurons after brain injury (Figure 4). If all neurons are forced to express "low-fitness" fingerprints, such unfit neurons are not removed by apoptosis. Our analysis has shown that damage-exposed cells are specifically eliminated around proliferating zones, where de novo neurogenesis is taking place (Figures 1E–1J). We propose a model in which newly born cells are favored over unfit damaged neurons to reconstitute the adult brain based on Flower fitness fingerprints (Figure 4K). One possibility is that neurons partially damaged and/or displaced by the injury upregulate $\text{Flower}^{\text{LoseB}}$. A non-exclusive alternative is that newborn neurons play an active role in the elimination of less fit neurons.

Based on our data, cell death does not seem to be associated with physiologic adult neurogenesis (n = 20 left OLs), but further analysis with higher temporal resolution will be required to corroborate these results.

Is fitness-driven elimination of "old" cells that do not fit into regenerated tissues important in other regenerating tissue types? Our preliminary results show that specific Flower isoforms are induced in regenerating wing imaginal discs after cell ablation and in the adult midgut after irradiation (Figure S3) and [34].

Moreover, Darwinian-like cell selection could play a role during liver regeneration in mice. An initial study reported a striking increase in apoptosis of host hepatocytes immediately adjacent to transplanted progenitor cells, which can repopulate the liver [35]. It will be interesting to see if mouse Flower homologs [36] also play a role there.

We therefore propose that comparison of cellular fitness between damaged and intact tissue may be a common mechanism during regeneration and relevant for stem cell-based replacement therapies after injury.

EXPERIMENTAL PROCEDURES

Fly Stocks

The following fly stocks were used: *fweReporter(yfp_gfp_rfp)* [32], *fweReporter (myc_HA_flag)* [32] (a gift from H. Bellen), *GMR-Gal4, fweReporter (yfp_gfp_rfp)/Cyo; MKRS/TM6b; elav-Gal4; Gal80ts;* $\textit{UASbsk}^{\textit{DN}}$*; UASpuc; TRE::gfp* [31], *UASlacz; 10xStat92E-DGFP* [37, 38], *exp-lacz; Diap-lacz* (a gift from B. Thompson), $\textit{UAS-fwe}^{\textit{LoseB}}$ [12], $\textit{UAS-fwe}^{\textit{ubi}}$ [12], $\textit{UAS-fwe}^{\textit{LoseA}}$ [12], *RNAi flower* (KK); $\textit{RNAi flower}^{\textit{LoseA/B}}$ [13], and *w1118; +; rnGal4, UASeiger, tubGal80ts* [28] (a gift from I. Hariharan).

Perma-Twin Labeling

The following stocks were crossed: *w; FRT40A, UAS-CD8-GFP, UAS-CD2-Mir; act-Gal4 UAS-flp/TM6B* and *w; FRT40A, UAS-CD2-RFP, UAS-GFP-Mir; Gal80ts/TM6B.* Labeling was activated by shifting of 5-day-old adult flies from 18°C to 29°C for 24 hr, followed by traumatic brain injury.

RNAi Experiments

elav-Gal4/Cyo; Gal80ts/TM6B flies were crossed to *UASgene* or *UASRNAi-gene* lines at 18°C. Adults 3 to 4 days old were shifted for 5 days to 29° and then subjected to brain injury.

Penetrating Traumatic Brain Injury

Flies were immobilized on a CO_2 diffusion pad, and the medulla of the OL was injured with a thin sterile filament (diameter 0.1 mm; Fine Science Tools) [12]. Flies were allowed to recover for 1 hr at room temperature and were then shifted to 29°C. Comparable lesions (55–65 μm) were achieved by depth indicators on the perforating filament. Lesions were identified under the microscope based on pigment traces (lateral damage) deposited at the end of the needle tract, TUNEL staining, and brain morphology (DAPI).

Brain Dissection and Immunostaining

Brains were prepared as described previously [20]. The following antibodies were used: rat anti-Elav (1:50; Developmental Studies Hybridoma Bank); monoclonal rat anti-HA (1:500; Roche), polyconal anti-Myc-tag (1:50; Cell Signaling), $\text{anti-Flower}^{\text{ubi}}$ (1:30) [12] (in combination with Biotin-Streptavin amplification), and mouse anti-Serpent (1:200; a gift from J. Pastor-Pareja). TUNEL staining (Roche) was performed according to the supplier's protocol. Sections of OLs 50-μm thick were scanned and quantified for TUNEL-positive cells. Confocal images were acquired with a Leica SP8 microscope.

Statistical Analysis

First, an ANOVA model was fitted to log-transformed values of the apoptotic cell counts and validated via Tukey-Anscombe plot and QQ plot of the

Figure 4. Elimination of Unfit Tissue Is Mediated by Comparison of Neuronal Fitness

(A–H) Cell death (TUNEL; white) in laterally injured right or uninjured left OLs 72 hr AD. Dashed arrows indicate the area of injury.

(B–H) Merged images with DAPI to visualize cell nuclei.

(I and J) Quantification of apoptotic cells 24 hr (I) and 72 hr (J) AD in injured right OLs and undamaged left OLs. Statistical significance is based on ANOVA. Genotypes were compared to *UASlacz* controls. ***p < 0.001. Bold lines show the median, and the boxed area represents 25% and 75% quantiles. Note the logarithmic scale. n, the number of OLs, is plotted below.

(K) Model for tissue replacement during adult brain regeneration in *Drosophila*. Direct fitness comparison of impaired neurons (red) after injury and newly generated and intact neurons (green) via Flower fitness fingerprints drives elimination of unfit neurons.

Scale bars represent 20 μm.

residuals. Subsequently, p values were calculated by comparison of experimental genotypes with the control genotype (*UASlacz*) and were corrected for multiple testing with Holm's method [39].

SUPPLEMENTAL INFORMATION

Supplemental Information includes Supplemental Experimental Procedures and three figures and can be found with this article online at http://dx.doi.org/10.1016/j.cub.2015.02.014.

AUTHOR CONTRIBUTIONS

E.M. and C.R. conceived and performed the experiments. P.M. and Y.-F.M. repeated some of the experiments and controls. E.M. and C.R. wrote the paper.

ACKNOWLEDGMENTS

We thank Dr. Alain Hauser for help with statistical analysis; H. Bellen, B. Thompson, K. Basler, I. Hariharan, and the Bloomington stock center for providing flies; and the Hybridoma Bank for antibodies. Research in this paper was supported by the University of Bern (C.R.) and grants of the European Research Council, the Swiss National Science Foundation, and the Josef Steiner Cancer Research Foundation to E.M.

Received: November 10, 2014
Revised: January 12, 2015
Accepted: February 2, 2015
Published: March 5, 2015

REFERENCES

1. Morata, G., and Ripoll, P. (1975). Minutes: mutants of *drosophila* autonomously affecting cell division rate. Dev. Biol. *42*, 211–221.
2. Raff, M.C. (1992). Social controls on cell survival and cell death. Nature *356*, 397–400.
3. Moreno, E., Basler, K., and Morata, G. (2002). Cells compete for decapentaplegic survival factor to prevent apoptosis in *Drosophila* wing development. Nature *416*, 755–759.
4. Moreno, E., and Basler, K. (2004). dMyc transforms cells into super-competitors. Cell *117*, 117–129.
5. de la Cova, C., Abril, M., Bellosta, P., Gallant, P., and Johnston, L.A. (2004). *Drosophila* myc regulates organ size by inducing cell competition. Cell *117*, 107–116.
6. Bondar, T., and Medzhitov, R. (2010). p53-mediated hematopoietic stem and progenitor cell competition. Cell Stem Cell *6*, 309–322.
7. Sancho, M., Di-Gregorio, A., George, N., Pozzi, S., Sánchez, J.M., Pernaute, B., and Rodríguez, T.A. (2013). Competitive interactions eliminate unfit embryonic stem cells at the onset of differentiation. Dev. Cell *26*, 19–30.
8. Clavería, C., Giovinazzo, G., Sierra, R., and Torres, M. (2013). Myc-driven endogenous cell competition in the early mammalian embryo. Nature *500*, 39–44.
9. Martins, V.C., Busch, K., Juraeva, D., Blum, C., Ludwig, C., Rasche, V., Lasitschka, F., Mastitsky, S.E., Brors, B., Hielscher, T., et al. (2014). Cell competition is a tumour suppressor mechanism in the thymus. Nature *509*, 465–470.
10. Vermeulen, L., Morrissey, E., van der Heijden, M., Nicholson, A.M., Sottoriva, A., Buczacki, S., Kemp, R., Tavaré, S., and Winton, D.J. (2013). Defining stem cell dynamics in models of intestinal tumor initiation. Science *342*, 995–998.
11. Tamori, Y., Bialucha, C.U., Tian, A.G., Kajita, M., Huang, Y.C., Norman, M., Harrison, N., Poulton, J., Ivanovitch, K., Disch, L., et al. (2010). Involvement of Lgl and Mahjong/VprBP in cell competition. PLoS Biol. *8*, e1000422.
12. Rhiner, C., López-Gay, J.M., Soldini, D., Casas-Tinto, S., Martín, F.A., Lombardía, L., and Moreno, E. (2010). Flower forms an extracellular code that reveals the fitness of a cell to its neighbors in Drosophila. Dev. Cell *18*, 985–998.
13. Merino, M.M., Rhiner, C., Portela, M., and Moreno, E. (2013). "Fitness fingerprints" mediate physiological culling of unwanted neurons in *Drosophila*. Curr. Biol. *23*, 1300–1309.
14. Vincent, J.P., Fletcher, A.G., and Baena-Lopez, L.A. (2013). Mechanisms and mechanics of cell competition in epithelia. Nat. Rev. Mol. Cell Biol. *14*, 581–591.
15. Sánchez Alvarado, A., and Tsonis, P.A. (2006). Bridging the regeneration gap: genetic insights from diverse animal models. Nat. Rev. Genet. *7*, 873–884.
16. Tornini, V.A., and Poss, K.D. (2014). Keeping at arm's length during regeneration. Dev. Cell *29*, 139–145.
17. Carmichael, S.T. (2006). Cellular and molecular mechanisms of neural repair after stroke: making waves. Ann. Neurol. *59*, 735–742.
18. Loane, D.J., and Faden, A.I. (2010). Neuroprotection for traumatic brain injury: translational challenges and emerging therapeutic strategies. Trends Pharmacol. Sci. *31*, 596–604.
19. Andriessen, T.M., Jacobs, B., and Vos, P.E. (2010). Clinical characteristics and pathophysiological mechanisms of focal and diffuse traumatic brain injury. J. Cell. Mol. Med. *14*, 2381–2392.
20. Fernández-Hernández, I., Rhiner, C., and Moreno, E. (2013). Adult neurogenesis in *Drosophila*. Cell Rep. *3*, 1857–1865.
21. Tanaka, E.M., and Reddien, P.W. (2011). The cellular basis for animal regeneration. Dev. Cell *21*, 172–185.
22. Robinow, S., and White, K. (1988). The locus elav of *Drosophila melanogaster* is expressed in neurons at all developmental stages. Dev. Biol. *126*, 294–303.
23. Bergantiños, C., Corominas, M., and Serras, F. (2010). Cell death-induced regeneration in wing imaginal discs requires JNK signalling. Development *137*, 1169–1179.
24. Noselli, S., and Agnès, F. (1999). Roles of the JNK signaling pathway in *Drosophila* morphogenesis. Curr. Opin. Genet. Dev. *9*, 466–472.
25. Thompson, B.J., and Cohen, S.M. (2006). The Hippo pathway regulates the bantam microRNA to control cell proliferation and apoptosis in *Drosophila*. Cell *126*, 767–774.
26. Grusche, F.A., Degoutin, J.L., Richardson, H.E., and Harvey, K.F. (2011). The Salvador/Warts/Hippo pathway controls regenerative tissue growth in *Drosophila* melanogaster. Dev. Biol. *350*, 255–266.
27. Shaw, R.L., Kohlmaier, A., Polesello, C., Veelken, C., Edgar, B.A., and Tapon, N. (2010). The Hippo pathway regulates intestinal stem cell proliferation during *Drosophila* adult midgut regeneration. Development *137*, 4147–4158.
28. Smith-Bolton, R.K., Worley, M.I., Kanda, H., and Hariharan, I.K. (2009). Regenerative growth in *Drosophila* imaginal discs is regulated by Wingless and Myc. Dev. Cell *16*, 797–809.
29. Cordero, J.B., Stefanatos, R.K., Scopelliti, A., Vidal, M., and Sansom, O.J. (2012). Inducible progenitor-derived Wingless regulates adult midgut regeneration in *Drosophila*. EMBO J. *31*, 3901–3917.
30. Karpowicz, P., Perez, J., and Perrimon, N. (2010). The Hippo tumor suppressor pathway regulates intestinal stem cell regeneration. Development *137*, 4135–4145.
31. Chatterjee, N., and Bohmann, D. (2012). A versatile ΦC31 based reporter system for measuring AP-1 and Nrf2 signaling in *Drosophila* and in tissue culture. PLoS ONE *7*, e34063.
32. Yao, C.K., Lin, Y.Q., Ly, C.V., Ohyama, T., Haueter, C.M., Moiseenkova-Bell, V.Y., Wensel, T.G., and Bellen, H.J. (2009). A synaptic vesicle-associated Ca2+ channel promotes endocytosis and couples exocytosis to endocytosis. Cell *138*, 947–960.
33. Freeman, M.R. (2014). Signaling mechanisms regulating Wallerian degeneration. Curr. Opin. Neurobiol. *27*, 224–231.

34. Merino, M.M., Rhiner, C., Lopez-Gay, J.M., Buechel, D., Hauert, B., and Moreno, E. (2015). Elimination of unfit cells maintains tissue health and prolongs lifespan. Cell *160*, 461–476.

35. Oertel, M., Menthena, A., Dabeva, M.D., and Shafritz, D.A. (2006). Cell competition leads to a high level of normal liver reconstitution by transplanted fetal liver stem/progenitor cells. Gastroenterology *130*, 507–520, quiz 590.

36. Petrova, E., López-Gay, J.M., Rhiner, C., and Moreno, E. (2012). Flower-deficient mice have reduced susceptibility to skin papilloma formation. Dis. Model. Mech. *5*, 553–561.

37. Bach, E.A., Ekas, L.A., Ayala-Camargo, A., Flaherty, M.S., Lee, H., Perrimon, N., and Baeg, G.H. (2007). GFP reporters detect the activation of the *Drosophila* JAK/STAT pathway in vivo. Gene Expr. Patterns *7*, 323–331.

38. Classen, A.K., Bunker, B.D., Harvey, K.F., Vaccari, T., and Bilder, D. (2009). A tumor suppressor activity of *Drosophila* Polycomb genes mediated by JAK-STAT signaling. Nat. Genet. *41*, 1150–1155.

39. Holm, S. (1979). A simple sequentially rejective multiple test procedure. Scand. J. Stat. *6*, 65–70.

Current Biology

Supplemental Information

Brain Regeneration in *Drosophila* Involves Comparison of Neuronal Fitness

Eduardo Moreno, Yuniel Fernandez-Marrero, Patricia Meyer, and Christa Rhiner

Supplemental Data

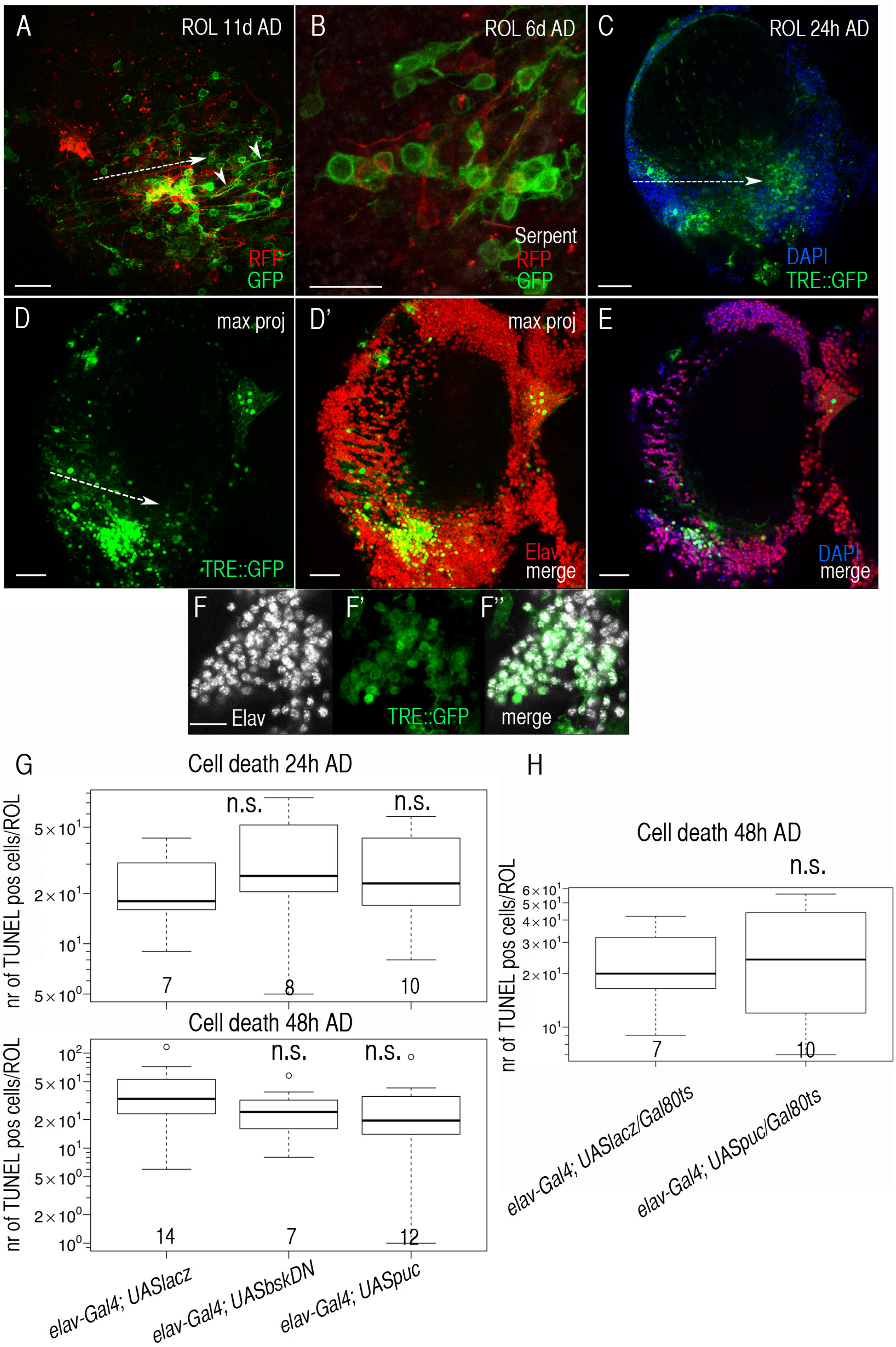

Figure S1

Figure S1, related to Figure1: Inhibition of JNK activity does not prevent cell death after brain injury

(A) Newly generated tissue marked by perma-twin labeling (GFP and RFP) shows neuronal morphology 11 days after damage (AD) in the right optic lobe (ROL). Neurons extend long axons (arrowheads) to proper target area (lobular plate). Dashed arrow marks lesion site.

(B) Perma-twin labeled tissue (GFP/RFP) does not express the hemocyte marker Serpent (white) 6 days AD [S1].

(C) JNK signaling is activated in large areas of the injured right optic lobe (ROL) based on *TRE::gfp* reporter activity.

(D-F) JNK pathway activity is present in numerous neurons 24h AD. Panels D show projections of numerous layers, E and F (inset) represent single layers. Neurons are stained with Elav (red, D, E and white, F); cell nuclei are shown in blue (E).

(G) Quantification of cell death 24h and 48h AD. JNK activity was suppressed in neurons throughout development. No statistical significance based on ANOVA (n.s.) (48h AD: $p = 0.35$ for $UASbsk^{DN}$; $p = 0.17$ for *UASpuc*). *$p < 0.1$.

(H) Quantification of cell death 48h AD. Inhibition of JNK activity with *UASpuc* was activated specifically in the adult nervous system. No statistical significance based on ANOVA (n.s.) ($p = 0.98$ for *UASpuc*). Bold lines show median, boxed area represents 25 and 75% quantiles. Note the logarithmic scale. n = nr of optic lobes are plotted below. Scale bars are 10 μm (B, F) and 20 μm for A, C-E).

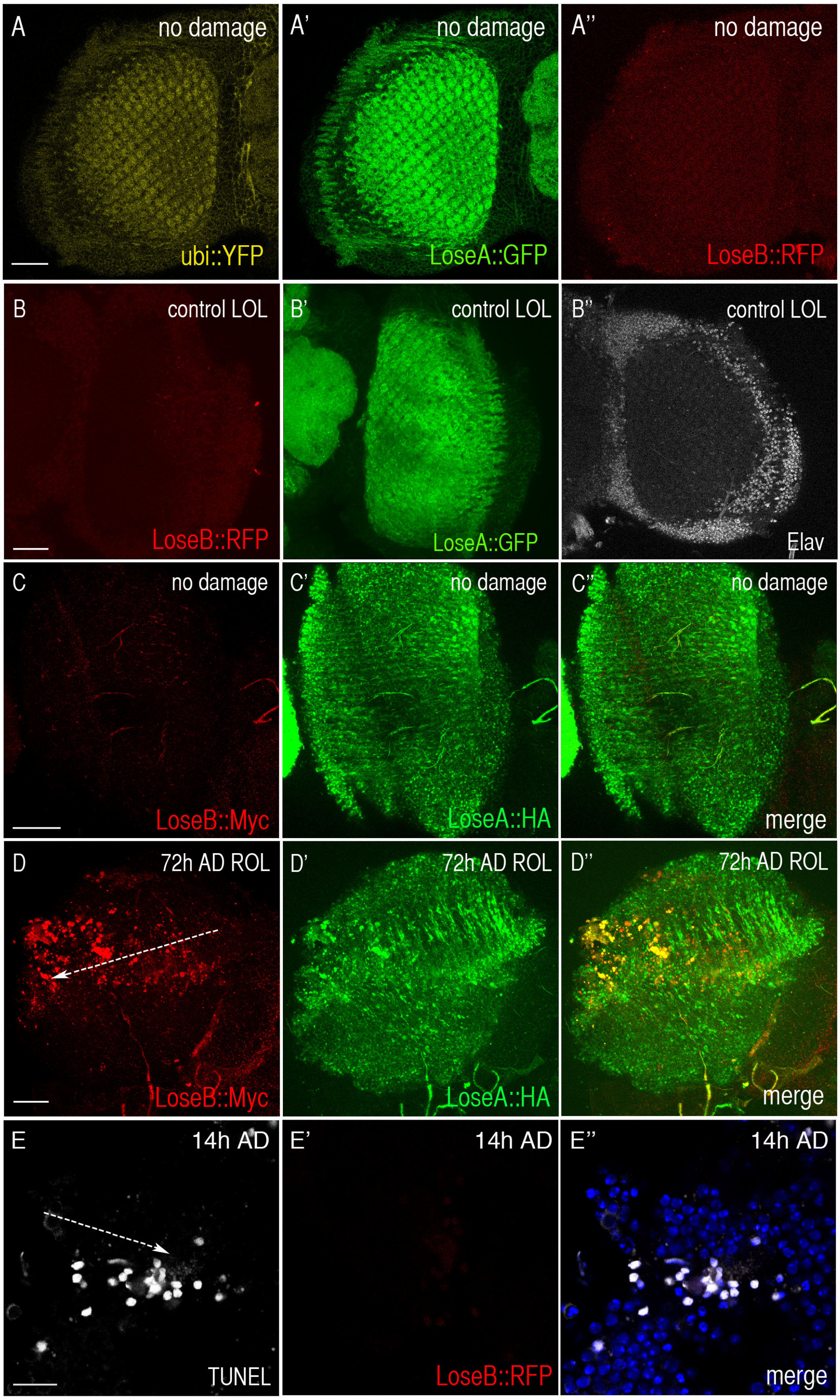
A
no damage
ubi::YFP
A'
no damage
LoseA::GFP
A''
no damage
LoseB::RFP
B
control LOL
LoseB::RFP
B'
control LOL
LoseA::GFP
B''
control LOL
Elav
C
no damage
LoseB::Myc
C'
no damage
LoseA::HA
C''
no damage
merge
D
72h AD ROL
LoseB::Myc
D'
72h AD ROL
LoseA::HA
D''
72h AD ROL
merge
E
14h AD
TUNEL
E'
14h AD
LoseB::RFP
E''
14h AD
merge

Figure S2

Figure S2, related to Figure 2: Damage-modulated expression of Flower isoforms

(A) Expression of $Flower^{ubi}$ (YFP), $Flower^{LoseB}$ (RFP) and $Flower^{LoseA}$ (GFP) in the optic lobe of the adult brain based on a translational *flower* reporter.

(B) Expression of $Flower^{LoseB}$ (RFP) and $Flower^{LoseA}$ (GFP) in the undamaged control left optic lobe (LOL). Neurons (Elav+) are shown in white.

(C and D) Flower isoform expression in undamaged (C) and apically damaged optic lobes (arrow, through head cuticle) (D) as evidenced by antibody staining against HA-tagged $Flower^{LoseA}$ (green) and Myc-tagged $Flower^{LoseB}$ (red) based on a translational *flower* reporter 72h after damage (AD).

(E) Apoptotic cells (TUNEL, white) 14h AD do not show upregulation of $Flower^{LoseB::RFP}$ (red). Arrow marks injury site.

Scale bars are 20 μm (A-D) and 10 μm (E).

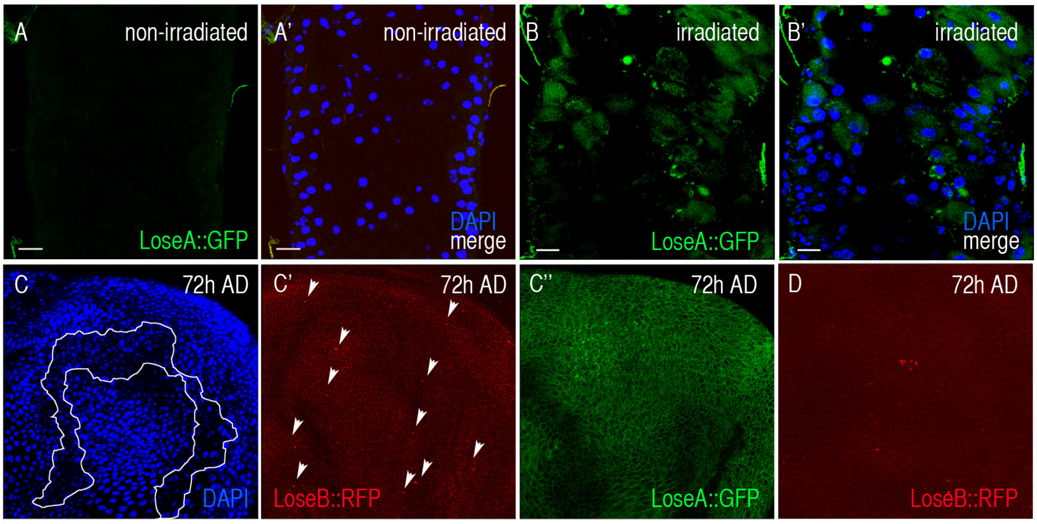

Figure S3

Figure S3, related to Figure 2: Flower fitness fingerprints in the regenerating gut.

(A) Flower^{LoseA} (GFP) expression in the non-irradiated midgut. Nuclei are shown in blue (DAPI).

(B) Flower^{LoseA} (GFP) is upregulated in the midgut 24h after irradiation.

(C) Flower^{LoseB} expression (RFP, arrowheads) 72h after wing disc ablation with *rnGal4, UASeiger, tubGal80^{ts}* in the apoptotic region (marked in white) containing numerous apoptotic corpses (small nuclei, DAPI, blue).

(D) Foci of Flower^{LoseB}-expressing cells (RFP) 72h after wing disc ablation with *rnGal4, UASeiger, tubGal80^{ts}*.

Scale bars are 20 μm.

Supplemental Experimental Procedures

Midgut irradiation. 2-3 days old adult flies were subjected to 2 x 10^{-2} J of UV irradiation with a UV Stratalinker 2400 machine and analyzed for Flower isoform expression 24h later.

Wing pouch ablation. w^{1118}; +; *rnGal4, UASeiger, tubGal80*^{ts} flies were crossed to *fweReporter(yfp_gfp_rfp)* flies at 18°C. Larvae were shifted for 20h to 29°C to induce wing disc ablation and then placed back to 18°C to allow regeneration.

Supplemental References

[S1] Lebestky T, Chang T, Hartenstein V, Banerjee U (2000). Specification of Drosophila hematopoietic lineage by conserved transcription factors. Science. 288:146-149.
